# Maternal Obesity, Inflammation, and Developmental Programming

**DOI:** 10.1155/2014/418975

**Published:** 2014-05-20

**Authors:** Stephanie A. Segovia, Mark H. Vickers, Clint Gray, Clare M. Reynolds

**Affiliations:** Liggins Institute and Gravida, National Centre for Growth and Development, University of Auckland, Auckland 1142, New Zealand

## Abstract

The prevalence of obesity, especially in women of child-bearing age, is a global health concern. In addition to increasing the immediate risk of gestational complications, there is accumulating evidence that maternal obesity also has long-term consequences for the offspring. The concept of developmental programming describes the process in which an environmental stimulus, including altered nutrition, during critical periods of development can program alterations in organogenesis, tissue development, and metabolism, predisposing offspring to obesity and metabolic and cardiovascular disorders in later life. Although the mechanisms underpinning programming of metabolic disorders remain poorly defined, it has become increasingly clear that low-grade inflammation is associated with obesity and its comorbidities. This review will discuss maternal metainflammation as a mediator of programming in insulin sensitive tissues in offspring. Use of nutritional anti-inflammatories in pregnancy including omega 3 fatty acids, resveratrol, curcumin, and taurine may provide beneficial intervention strategies to ameliorate maternal obesity-induced programming.

## 1. Introduction

The prevalence of obesity in both developed and developing countries has been steadily increasing over the past 40 years [1–3]. Consequently, obesity and its associated comorbidities are a significant concern, in terms of global public health and public health spending. Depending on the population, the prevalence of obesity (body mass index ≥ 30 kg/m^2^) in women of reproductive age can be as high as 34% [3]. Obesity during pregnancy is of major concern due to the well-characterized risk factors to both the mother and her offspring. These can include, but are not limited to, maternal and fetal death, preeclampsia, gestational diabetes, and congenital abnormalities [4]. In addition, epidemiological evidence and data derived from animal models have demonstrated that maternal obesity has long-term consequences for offspring, predisposing or “programming” them to the development of metabolic disease in adulthood [5]. It has become increasingly clear that metabolic disease is associated with a state of chronic low-grade inflammation [6]. Inflammation has received extensive attention recently because of its association with several diseases, including cancer, diabetes, and obesity—it is a tightly regulated process—deviations from this process present a significant health risk because unresolved inflammation can compromise tissue function. In human pregnancies, maternal obesity is associated with metabolic inflammation, characterized by elevated adipose tissue and systemic proinflammatory cytokine levels and adipose tissue macrophage accumulation [7, 8]. Additionally, these changes extend to the placenta, suggesting that maternal obesity exposes the fetus to an inflammatory environment during development [9]. Thus, in the context of developmental programming, early life exposure to metabolic inflammation may represent a key mechanism by which developmentally programmed phenotypes may manifest later in life. For example, in animal models, maternal obesity has been shown to induce fetal inflammation which can result in promotion of adipogenesis and increased adiposity in offspring [10]. The critical windows of innate immune vulnerability during prenatal and neonatal maturation are when developmental programming and the trajectory for childhood and adult inflammatory responses are largely established. Clearly, there is a need for targeted intervention strategies to ameliorate and reduce the adverse effects of maternal obesity on offspring health outcomes during later life. This review will discuss maternal obesity related inflammation as a mechanism of developmental programming of metabolic disorders in offspring and the potential of intervention strategies.

## 2. The Developmental Origins of Health and Disease 

Barker first suggested that the fetal environment may have an effect on the development of disease in adulthood, known as the fetal origins of adult disease (FOAD) hypothesis [11]. Epidemiological evidence from UK birth records indicated a geographical correlation between high rates of infant mortality and adult ischaemic heart disease [12]. They hypothesized that maternal undernutrition resulted in fetal programming which caused permanent alterations in the structure, function, homeostatic pathways, and/or metabolism of the developing offspring, predisposing them to disease later in life. Since these initial observations, the concept has evolved into the developmental origins of health and disease (DOHaD) hypothesis, which describes the process by which an environmental stimulus, including altered nutrition, during a critical period of development can program alterations in organogenesis, tissue development, and metabolism, predisposing offspring to metabolic and cardiovascular dysfunction during adulthood [13–15]. These effects can be amplified in the setting of a poor postnatal diet [16].

In today's society, maternal obesity is a more prevalent and emerging cause for concern. Considerable epidemiological evidence demonstrates that maternal obesity is a predictor for development of obesity, type 2 diabetes, and cardiovascular disease in offspring [17–19]. Mechanistic studies in human cohorts are challenging due to the number of potential postnatal confounders and the time course required for prospective studies and thus remain largely observational. Therefore, animal studies have become the primary tool for investigating the myriad of potential mechanisms underlying the developmental programming paradigm. Maternal obesity-induced developmental programming has been validated in mouse, rat, sheep, and nonhuman primate models and has been shown to affect numerous metabolic pathways culminating in a metabolic syndrome like phenotype [20–23].

There is an increasing body of evidence demonstrating the capability to ameliorate or reverse programming by targeted interventions during specific periods of developmental plasticity [24–26]. A particular focus has been on the adipokine leptin (a proinflammatory signal in adipose tissue) as a mediator of programmed changes in the regulation of appetite and metabolism. Obese individuals exhibit higher circulating levels of leptin, contributing to a state of leptin resistance, which further perpetuates obesity, inflammation, and metabolic disease [27]. Leptin levels are known to be elevated in pregnancies complicated with enhanced inflammatory processes in the placentae [9, 28]. Of note, maintenance of a critical leptin level during early development facilitates the normal maturation of tissues and signaling pathways involved in metabolic homeostasis. In rats, maternal undernutrition results in neonatal hypoleptinemia—leptin administration to these neonates reverses maternal undernutrition-induced metabolic programming in adult female offspring [29]. We have also shown that preweaning growth hormone treatment in a rat model of undernutrition reverses programmed hypertension, obesity, and inflammatory profiles in adult offspring [26, 30]. In rats, supplementation with docosahexaenoic acid (DHA) in the setting of maternal undernutrition has also been shown to protect offspring against later metabolic dysfunction [31], but data in the setting of maternal obesity are less clear. In rat models of protein restriction, dietary cofactors, including folate and glycine, have also been shown to reverse postnatal metabolic and cardiovascular abnormalities in offspring [32–34].

Taken together, these studies suggest that programming effects can be prevented by early intervention strategies. To date, the majority of developmental programming studies are primarily descriptive and the underlying mechanisms, particularly as regards the inflammasome, of how maternal obesity impacts upon early life development and subsequent adult disease phenotypes are not well understood. Elucidating the mechanisms of maternal obesity-induced developmental programming is of utmost importance and may allow for application of therapeutic and/or nutritional interventions to minimize adverse programming effects in offspring.

## 3. Adipose Tissue Dysfunction in Obesity 

Historically, white adipose tissue (WAT) was viewed as an inert energy storage depot. However, it is now appreciated as a major endocrine organ which contributes to metabolic homeostasis. Adipose tissue is composed of not only multiple cell types, mainly adipocytes (fat cells), but also the stromal vascular fraction (SVF), which includes preadipocytes, fibroblasts, endothelial cells, and immune cells [35]. Adipose tissue secretes a broad range of bioactive factors, collectively referred to as adipokines [36]. Adipokines have a range of essential physiological roles, including adipocyte differentiation, glucose and lipid metabolism, satiety, immune regulation, cardiovascular function, and neuroendocrine function [37]. Aberrant regulation of adipokine secretion has been shown to mediate cross talk with other organs and contribute to the development of obesity-induced comorbidities such as insulin resistance and metabolic syndrome [38–40]. The complex adipokine profile is still not fully understood, with novel adipokines still being identified [41, 42]. Additionally, it is important to note that the study of adipose tissue dysfunction is confounded by the wide range of etiologies which include genetics, environment, and now early life stressors such as maternal obesity.

In healthy individuals, the adipose tissue is composed of mainly preadipocytes and adipocytes, with few inflammatory leukocytes. With obesity, the composition, phenotype, and function of adipose tissue are disrupted [43–45]. Persistent excess energy intake causes adipocytes to undergo hypertrophy (increased adipocyte volume) in attempt to meet the increased energy storage needs [46]. Adipocyte hypertrophy can contribute to further complications including hypoxia, adipocyte necrosis, chemokine secretion, and compromised regulation of fatty acid flux [47]. Hypertrophied cells alter the balance of adipose tissue-derived cytokines and adipokines to a proinflammatory state, acting as a critical factor linking obesity to the pathogenesis of metabolic disease in both mother and offspring [48, 49]. Inflammatory mediators, including C-reactive protein (CRP), interleukin-6 (IL-6), IL-1*β*, and tumour necrosis factor *α* (TNF*α*), are systemically elevated in obesity in animal and human models [50–52]. Additionally, as the adipose tissue expands, the blood supply becomes inadequate and hypoxia occurs [53]. This contributes to further cellular dysfunction in adipocytes, including downregulation of adiponectin mRNA expression and induction of endoplasmic reticulum stress, which can further exacerbate the inflammatory state [35, 53].

A hallmark of adipose tissue inflammation is the infiltration of immune cells including monocytes/macrophages, neutrophils, B lymphocytes, and T lymphocytes [54–56]. Macrophages are phagocytic cells, which act to engulf and digest pathogens and cellular debris. Adipose tissue macrophage infiltration has recently emerged as a major contributor of inflammatory mediators contributing to dysfunction in obesity after seminal publications by Xu et al. and Weisburg et al. in 2003. Xu et al. showed that macrophage-specific genes including monocyte chemoattractant protein-1 (MCP-1), macrophage inflammatory protein-1*α* (MIP-1*α*), CD11b, F4/80, and CD68 were upregulated in adipose tissue of diet-induced obese mice. Interestingly, this preceded development of hyperinsulinemia and treatment with the insulin sensitive drug rosiglitazone caused downregulation of these genes. Weisburg et al. analyzed the profile of 1304 body mass related transcripts, finding that 30% of the 100 most significantly correlated genes encoded genes which were characteristic of macrophages. Immunohistochemical analysis of multiple adipose depots showed a significant correlation between the percentage of F4/80 expression and adipocyte size and body mass. These results have since been corroborated in a number of studies [54, 57]. Surgical or diet-induced weight loss in obese individuals results in decreased MCP-1 gene expression and reductions in macrophage infiltration and inflammation [58, 59]. Additionally, macrophage activation appears to shift towards M2 (alternatively activated) over M1 (classically activated) status postgastric bypass surgery in morbidly obese individuals, contributing to a less inflammatory phenotype [60].

## 4. Metabolic Inflammation as a Programming Mechanism 

Interest in the developmental origins of obesity and its associated metabolic sequelae has grown in recent years. There is evidence to support a number of potential mechanisms, including programming of offspring appetite, gene expression, and functional changes to adipose tissue [61]. These conditions are linked by the activation of a number of inflammatory pathways, including the NLR family, pyrin domain containing 3 (NLRP3) inflammasome, peroxisome proliferator-activated receptors (PPAR) signaling, and nuclear factor-*κ*B (NF-*κ*B) pathway [62–64].

Classical inflammation is the body's process of responding to injury or infection to restore homeostasis [65]. However, in obesity, the inflammatory response, which has been coined “metainflammation,” is chronic and is on a lower scale than the typical classic inflammatory response. The persistent state of chronic low-grade inflammation induced by obesity is characterized by abnormal cytokine production, an altered adipokine profile, and activation of inflammatory pathways [6]. The role of chronic low-grade inflammation in obese mothers has become an emerging focus in the developmental programming field. Our group have demonstrated that unbalanced maternal nutrition results in metainflammation in the mother and programs inflammation in offspring tissues [30, 66, 67]. However, current understanding of how these pathways are activated in the context of developmental programming remains poorly defined. Of note, most studies have characterised the programmed offspring as adults when the phenotype is already manifested. There is now strong evidence that early changes in inflammatory markers can be predictors of later metabolic and cardiovascular disease; thus evaluation of offspring inflammatory profiles at early stages of development may provide a useful biomarker for later life metabolic adversity [68].

In rodents, maternal immune activation during pregnancy with an immunostimulant such as lipopolysaccharide (LPS) has been shown to modify the immune response of offspring [69, 70]. These offspring exhibit a more proinflammatory macrophage (M1) phenotype and enhanced IL-1*β* production upon immune challenge in adulthood. Similarly, a state of maternal obesity is linked to an enhanced inflammatory response in offspring. Challier et al. observed macrophage accumulation and increased expression of proinflammatory cytokine expression in placenta from obese women compared to those from lean women [9]. Infiltrating macrophages have the capability to secrete inflammatory cytokines into the maternal or fetal systemic circulation. It is speculated that this is a contributing mechanism for the programmed alterations in offspring metabolism associated with increased adiposity and insulin resistance. The placenta transports free fatty acids from the maternal circulation and transports them for uptake by the fetal liver, where they are esterified and released as triglycerides into the circulation [71]. This has implications for fetal growth in humans, where associations between increased maternal triglycerides and macrosomia (large for gestational age) in offspring have been reported [72]. Zhu et al. observed elevated free fatty acids, cholesterol, and triglycerides in fetal circulation from obese ewes which were accompanied by upregulation of toll-like receptor 4 (TLR4), NF-*κ*B, and JNK signalling in cotyledonary tissue [73]. These findings suggest greater fatty acid uptake by the placenta, which can cause activation of inflammatory pathways in the placenta. Therefore, the state of chronic low-grade inflammation in pregravid obesity persists in pregnancy and contributes to an inflammatory environment for the developing fetus.

## 5. Programmed Effects of Maternal Obesity on Metabolic Function in Offspring

In animal models of diet-induced obesity, high fat feeding during pregnancy programs features of the metabolic syndrome, independent of environmental factors and postnatal diet [74, 75]. In humans, a high BMI before pregnancy and during early pregnancy is predictive for having a high birth weight baby, with these babies being at higher risk of developing the features of metabolic syndrome [76, 77]. In contrast, maternal obesity is also found to be associated with an increased incidence of intrauterine growth restriction (IUGR) in humans, with supporting evidence from animal work, underscoring the complexity of the maternal obesity paradigm [78, 79]. Our group and the work of others have shown that rodent models provide strong evidence for tissue specific impairments in offspring from obese mothers [80–84]. Impairments to metabolically critical or insulin sensitive tissues, especially adipose tissue, pancreas, liver, and skeletal muscle, may have profound effects on the development of insulin resistance and type 2 diabetes in offspring.

### 5.1. Adipose Tissue

Adipogenesis is the process of the development of stem cell precursors into adipocytes and largely occurs during the late gestation and early postnatal life in humans [85]. This process is sensitive to* in utero* conditions, such as a deficient or excess nutrient supply. Turnover of adipose cells in adulthood is low, with adipocyte number leveling off in adulthood [86]. This underscores the importance of the* in utero* environment and early postnatal life in a predisposition to adult onset of obesity. Perturbation of adipogenesis, and therefore the development of the adipose tissue as a whole, can alter its functional metabolic properties. Maternal obesity can promote excess accumulation of body fat in offspring and predispose them to obesity during later life.

In a mouse model of maternal diet-induced obesity, 3-month-old offspring from obese dams exhibited adipocyte hypertrophy, reduced mRNA expression of *β*2- and *β*3-adrenoreceptors, and increased mRNA expression of PPAR-*γ*2, a key mediator of adipogenesis [21]. In a similar model in rats, offspring displayed increased adiposity in later life despite a normal birth weight, as well as a high percentage of large adipocytes in concomitance with enhanced PPAR-*γ* expression [20]. In sheep models, maternal overnutrition during the late gestational period programmed increased mRNA expression of PPAR-*γ*, lipoprotein lipase (LPL), adiponectin, and leptin in fetal perirenal fat [87]. These findings suggest maternal overnutrition and subsequent obesity may increase the lipogenic capacity of adipose tissue, promoting a shift from a thermogenic to lipid storage function, which could be a contributing cause of increased adiposity in offspring. While an increased fat mass in offspring may be acting as a compensatory mechanism to promote lipid storage rather than ectopic fat deposition, excessive adiposity causes aberrant inflammatory cytokine and adipokine regulation of the tissue and subsequently a metabolic syndrome-like phenotype. A recent study in mice by Murabayashi et al. demonstrated that offspring of mothers exposed to a high fat diet displayed increases in expression of TNF*α*, CD68, and MCP-1 and decreased GLUT4 mRNA expression, suggesting that maternal obesity may affect fetal insulin sensitivity by altering inflammatory processes [88].

### 5.2. Liver

In humans, the liver is the most metabolically complex organ, playing pivotal roles in whole body metabolism including regulation of glucose homeostasis, lipogenesis, detoxification, protein metabolism, cholesterol production, and bile production [89]. WAT is critical for storage of excess lipids, but in humans WAT development does not occur until the third trimester of pregnancy [90]. Therefore, it is postulated that maternal obesity results in excess exposure of the fetal liver to triglycerides, lipids, adipokines, and other factors, causing alterations in gene expression which upregulate lipogenesis and downregulate lipolysis, contributing to hepatic lipid accumulation and inflammation. In a number of animal models, maternal overnutrition is found to elevate triglyceride levels, increase inflammatory markers, and cause fatty livers in offspring [21, 91, 92]. Although the etiology of liver disease can vary, nonalcoholic fatty liver disease (NAFLD) linked to obesity and metabolic syndrome is currently one of the most common causes of adult chronic liver disease [93]. NAFLD refers to a progressive range of stages of pathologies caused by fat buildup within hepatocytes—simple fatty liver, nonalcoholic steatohepatitis (NASH), fibrosis, and cirrhosis [89]. In humans, NASH is associated with increased gene expression of inflammatory factors both locally in the liver and systemically [94, 95].

McCurdy et al. demonstrated the programming effects in response to a maternal high fat diet in nonhuman primates [23]. Interestingly, not all mothers receiving the high fat diet developed obesity and insulin resistance. However, when examined during the early third trimester of gestation, all offspring of high fat fed mothers demonstrated signs of NAFLD such as hepatic inflammation, triglyceride accumulation, and premature gluconeogenic gene activation. Elevated triglyceride levels were also observed in P30 and P180 offspring, and in addition, offspring had a 2-fold increase in body fat percentage. Collectively, these observations suggest that consumption of a chronic high fat diet can independently increase risk of offspring developing NAFLD. Similar results have been replicated in mice fed high fat diets during gestation and lactation. Increased fat depot weight, increased serum insulin, triglycerides, proinflammatory cytokines, and hepatic I*κ*B kinase phosphorylation were observed [96, 97]. In offspring of mice fed a high fat diet during only gestation (G), only lactation (L), or both (GL), hepatic steatosis was observed [98]. Expression of sterol regulatory element-binding protein-lc (SREBP-1c) expression was higher in G and GL offspring, indicating a stimulation of lipogenic gene transcription and fatty acid synthesis. Expression of GLUT-2 was reduced in G offspring, indicating impaired carbohydrate metabolism.

### 5.3. Skeletal Muscle

Comprising about 40–50% of body mass, skeletal muscle is the chief peripheral insulin responsive tissue, responsible for glucose and fatty acid uptake in response to insulin. Similar to adipose tissue, skeletal muscle displays enhanced inflammation in response to high fat feeding, including increases in proinflammatory macrophages and inflammatory gene expression [99, 100]. Chronic inflammation also occurs in insulin resistant skeletal muscle, displayed by increased macrophage infiltration and increased inflammatory cytokines [101, 102].

Alterations to the development of skeletal muscle can have physiological consequences for the offspring. Proper skeletal muscle development in the fetal period is of critical importance as there is no net increase in muscle fibre number after birth [103]. Skeletal muscle is also sensitive to an adverse* in utero *environment during development as it has a lower priority in nutrient partitioning compared to other organs including the brain, heart, and liver [104]. Additionally, changes to adipogenesis in the fetal skeletal muscle can induce increased number and size of intramuscular adipocytes, which can act in a paracrine fashion to contribute to insulin resistance later in life [64, 105].

In dams fed a cafeteria diet (palatable processed food with high fat and high sugar) during gestation lactation, offspring displayed increased adiposity at weaning, reduced muscle cross-sectional area, fewer muscle fibres, muscle atrophy, and fibre hypoplasia [106]. Functional impairments to muscle included intramuscular fat deposition and preferential fat accretion in muscle fibres. This was accompanied by an increase in muscle PPAR-*γ* expression, which was suggested as a compensatory response to maintain insulin sensitivity.

Work by Du et al. showed that maternal obesity resulted in low-grade inflammation which altered the commitment of mesenchymal stem cells in fetal muscle through mechanisms including inhibition of AMP-activated protein kinase (which promotes adipogenesis) and inflammation-induced epigenetic modifications via polycomb group proteins [107]. In the offspring of high fat fed ewes Akt phosphorylation (the main downstream insulin signalling pathway) and insulin receptor mRNA expression were reduced [64]. Additionally, inflammation was observed in skeletal muscle with upregulation of TLR2 and TLR4 expression, NF-*κ*B pathway (IKK phosphorylation), JNK pathway, and increased TNF*α* expression.

## 6. Anti-Inflammatory Strategies to Reverse Programming

Despite the evidence demonstrating maternal obesity and effects of inflammation in offspring, knowledge on the effectiveness of anti-inflammatory agents during pregnancy is minimal. Although health care professionals highly recommend weight loss to reduce the risk factors associated with obesity during pregnancy, women are likely to maintain prepregnancy lifestyle habits throughout pregnancy. Therefore, this avenue is of utmost importance as rates of obesity continue to increase and the long-term effects negative to offspring become more apparent.

### 6.1. Omega 3 Fatty Acids: Eicosapentaenoic Acid (EPA) and Docosahexaenoic Acid (DHA)

Polyunsaturated fatty acids (PUFAs) are a group of lipids which can modulate the immune system, alter the regulation of pro- and anti-inflammatory cells, and affect transcriptional regulation [108]. The two main families of PUFAs are omega 6 (n-6; linoleic acid (LA)) and omega 3 (n-3; alpha linolenic acid (ALA), eicosapentanoic acid (EPA), and docosahexanoic acid (DHA)) fatty acids [109]. EPA and DHA are highly associated with brain function [110]. In general, eicosanoids derived from n-6 PUFAs are more proinflammatory and immunoactive; eicosanoids derived from n-3 PUFAs are therefore considered more anti-inflammatory [108]. PPARs, a group of nuclear receptor proteins that act as transcription factors and are responsible for regulating the expression of genes involved in adipogenesis, inflammation, and lipid metabolism, can be activated by a diverse range of ligands, including omega 3 and 6 fatty acids [111]. The Western diet, which has also become increasingly predominant in developing countries, contains a significantly higher proportion of n-6 PUFA compared to n-3 PUFA [112]. Associations between low n-3 PUFA intake and increases in the incidence of obesity, cardiovascular disease, inflammatory diseases, and cancer have been an area of active research [113]. EPA and DHA have an antiobesogenic effect and can both reduce existing adiposity and prevent high fat induced obesity [114, 115]. The n-3 PUFAs have been documented to exert anti-inflammatory effects in the context of obesity by modulating adipose tissue, skeletal muscle, and hepatic function [116].* In vitro*, EPA stimulates glucose and fatty acid uptake in skeletal muscle cells by increasing expression of the transporters GLUT1 and CD36/FAT (fatty acid translocase) and increasing glucose oxidation [117]. In the adipose tissue of obese rats, n-3 PUFA modulates the secretion profile of adipokines and cytokines, decreasing secretion of proinflammatory cytokines including TNF*α* and IL-6 and reducing MCP-1 levels and adipose tissue macrophage infiltration, contributing to anti-inflammatory and insulin sensitizing effects [118]. Upon high fat feeding in rats, n-3 PUFAs increase fatty acid oxidation and inhibit lipogenesis in the liver, causing fatty acids to be preferentially oxidized rather than being stored [119]. The PPAR signalling pathway is implicated as a mechanism for the insulin sensitizing effects of EPA [120]. Neschen et al. conducted a study in which wildtype mice and PPAR-*α* knockout mice were fed an isocaloric high fat diet with or without additional fish oil [121]. Within wildtype mice, the fish oil supplemented group had improved hepatic insulin sensitivity. These effects were not seen within PPAR-*α* knockout mice, suggesting the insulin sensitizing effects are attributed to PPAR signalling. Despite the strong support of improvements by EPA and DHA to obesity and related insulin resistance in animal models, results in human clinical trials have been less consistent, as human trials are complicated by composition of n-3 PUFAs used, dosage, duration of administration, dietary and lifestyle habits, and other confounders [122]. Furthermore, the amnion, which surrounds the developing embryo, is sensitive to inflammatory modulation by EPA and DHA, likely partially mediated by PPAR-*γ* [123]. Explants treated with EPA, DHA, or a mixture had reduced IL-8 and IL-6 concentrations compared to untreated controls. When treated with a PPAR-*γ* agonist, IL-8 secretion was significantly decreased, yet this effect was partially reversed when treated with a PPAR-*γ* antagonist. Short-term supplementation with pioglitazone, an insulin sensitizing agent that stimulates PPAR-*γ*, to offspring from obese mothers attenuated the programmed obesity and insulin resistance associated with maternal obesity [124]. A study by Heerwagen et al., using Fat-1 transgenic mice (capable of converting endogenous n-6 PUFA to n-3 PUFA), demonstrated the potential to reduce inflammation associated with diet-induced obesity and improve metabolic outcomes in offspring [125]. Fat-1 mice were protected from adverse effects of a high fat diet, including adipose tissue macrophage accumulation and systemic increases in TNF*α*, IL-1*β*, IL-6, and MCP-1. Although there were no observed changes in inflammatory markers in the placenta, fetuses from high fat diet mothers showed minor growth restriction compared to mothers on a control diet, which has also been previously reported [79]. Additionally, although high fat fed mothers did not display hyperlipidemia when measured in late pregnancy, their offspring had increased lipid deposition in the fetal liver, which was reduced in offspring from Fat-1 high fat diet mothers. This underscores that birth weight may not be an accurate measure of fetal health, but rather other measures (e.g., hepatic lipid accumulation) may be more accurate. Adult wildtype male offspring from Fat-1 high fat diet mothers displayed less adiposity, hepatic lipid accumulation, adipose tissue macrophages, and insulin resistance, compared to offspring from high fat mothers. Collectively, these findings suggest that targeting inflammatory processes involved in maternal overnutrition and obesity may be beneficial in reversing or mitigating harmful programming effects on offspring in later life.

### 6.2. Resveratrol

Resveratrol is a stilbenoid (natural phenol) and phytoalexin naturally produced by some plants, such as Japanese knotweed and the skin of red grapes [126]. Resveratrol gained significant interest when it was proposed to be responsible for the beneficial cardiovascular effects of red wine, described as the French paradox [127]. Subsequent studies have shown resveratrol to have a multitude of health benefits, including cancer chemopreventive, antioxidant, antiplatelet, and estrogen modulatory and caloric restriction mimetic activities [128–131]. Resveratrol increases the expression of sirtuin 1 (SIRT1), a nicotinamide adenine dinucleotide (NAD+)-dependent deacetylase [132]. SIRT1 has been shown to modulate genes which regulate a number of biological processes including cell proliferation, apoptosis, gluconeogenesis, lipolysis, adipogenesis, and inflammation [133–136]. Resveratrol treatment results in activation of peroxisome proliferator-activated receptor gamma coactivator 1-alpha (PGC-1*α*) by SIRT1, which prevents development of diet-induced obesity and insulin resistance [137]. Resveratrol has been shown to attenuate TNF*α* induced MCP-1 gene expression and secretion in a dose-dependent manner in 3T3-L1 adipocytes [138]. These effects were not observed when adipocytes were treated with a NF-*κ*B inhibitor prior to resveratrol exposure, suggesting this effect was mediated by NF-*κ*B.

Resveratrol's antidiabetic activity, including improving insulin sensitivity and decreasing glucose, dyslipidemia, and adiposity, has been well documented in diet-induced and genetic diabetic animal models [139, 140]. Resveratrol's antidiabetic activity is at least partially mediated by AMP-activated protein kinase (AMPK), which is involved in regulating mitochondrial biogenesis, inducing fatty acid oxidation in the liver and muscle, increasing muscle glucose uptake, and inhibiting lipogenic activity, collectively resulting in increased insulin sensitivity [141]. In mice deficient in either the a1 or a2 catalytic subunit of AMPK, resveratrol does not significantly affect insulin sensitivity or glucose tolerance, implicating AMPK as a mechanism for resveratrol's effects [142]. Long-term intracerebrovascular infusion in high fat fed obese/diabetic mice has been shown to normalize hyperglycemia and improve hypoinsulinemia associated with NF-*κ*B activation [143]. These effects were independent of changes to body weight and food intake, suggesting a potential role of the central nervous system in resveratrol's antidiabetic activity.

The hypoglycemic effects of resveratrol are critical in avoiding diabetic neuropathies and damaging effects to organs. A consequence of diabetes during pregnancy is diabetic embryopathy, which is associated with oxidative stress and can disrupt normal organogenesis [144]. Embryos from diabetic dams had increased apoptosis and oxidative stress markers, but resveratrol administration to diabetic dams during pregnancy protected against these effects and also improved measures of embryonic development including weight, crown rump length, and somite number [145].

In rats, doses of up to 750 mg/kg/d during gestation did not result in fetal abnormalities or have adverse effects on placenta weight or litter size [146]. A recent study conducted by Roberts et al. assessed the role of resveratrol supplementation during pregnancy in nonhuman primates. Mothers were fed a Western-style diet (36% fat) with or without resveratrol supplementation. Resveratrol improved placental inflammatory markers (IL-1*β* and macrophage migration inhibitory factor), maternal and fetal hepatic triglyceride accumulation, uterine blood flow, and insulin sensitivity [147]. Resveratrol was detected in maternal plasma, demonstrating an ability to cross the placental barrier and exert effects on the fetus. Although resveratrol supplementation did not alter fetal body mass, there was a 42% increase in pancreas mass in the fetus, which was confirmed by immunohistochemistry. Furthermore, resveratrol was shown to increase uterine artery blood flow thereby increasing fetal weight in a murine model of fetal growth restriction [148]. There is also evidence that resveratrol improves the metabolic profile of offspring born growth restricted by reversing Akt mediated insulin resistance in liver and skeletal muscle [149]. Taken together, these findings suggest that resveratrol may improve the maternal and offspring metabolic profile in maternal overnutrition, obesity, and diabetic pregnancies. However, as the consequences of resveratrol treatment have significant effects on parameters such as pancreatic mass in offspring with unknown long-term effects, further studies in humans to determine adequate and safe therapeutic dosage and routes and frequency of administration are required.

### 6.3. Curcumin

Curcumin is a polyphenol responsible for the yellow pigment present in turmeric. Its use in traditional medicine is well known, but research now supports anti-inflammatory, antidiabetic, antioxidant, chemopreventive, and cardiovascular protective properties [150, 151]. Curcumin is pleiotropic, with the ability to interact with various targets and exert its effects through several mechanisms of action. Curcumin has been shown to reduce the inflammatory response through NF-*κ*B, suppressing its activation by inhibiting I*κ*K*α* kinase (IKK) activation [152, 153]. Curcumin decreases inflammation by acting as an agonist of PPAR-*γ*. In a rodent model of sepsis, intravenous administration of curcumin resulted in downregulation of TNF*α* and decreased markers of tissue damage. Administration of a PPAR-*γ* antagonist reversed these effects, confirming the decreased inflammation to be mediated via PPAR-*γ* [154]. Studies have also shown that curcumin may have beneficial anti-inflammatory effects for treatment of postoperative inflammation, acute respiratory distress syndrome, and inflammatory bowel disease [155–157]. In mouse models of obesity-induced insulin resistance, oral administration of curcumin has beneficial effects on the inflammatory response and decreased insulin sensitivity associated with high fat feeding [158, 159]. Increased adiponectin, decreased TNF*α* and MCP-1, reduced macrophage infiltration, attenuation of NF-*κ*B activation, and inhibition of lipogenic gene expression were also observed.

Although studies of the use of curcumin in pregnancy are lacking, its safety in humans has been demonstrated, with doses of up to 12 g/day being well tolerated and having low toxicity [160–162]. In a two-generation reproductive toxicity study in Wistar rats, there was no observed adverse effect level on reproductive performance in two successive generations, even in high doses of 10000 ppm (equivalent to 847.4 mg/kg body weight in F0 males) [163]. However,* in vitro* exposure of curcumin to mouse blastocysts during the early postimplantation stages had adverse effects in a dose-dependent manner [164]. Administration of pegylated curcumin (increased solubility) in mice had negative effects to reproductive functions, attributed to estrogen-mimicking or androgen-antagonizing properties [165]. These discrepancies are likely attributed variations in the route of administration. Curcumin is lipophilic and oral administration involves digestion, absorption, and metabolism in the liver, therefore reducing bioavailability at the target organ. Direct administration of curcumin* in vitro* does not accurately reflect physiologic conditions.

However the anti-inflammatory effects of curcumin have been shown to reverse ethanol-induced cognitive impairments in rat offspring by dampening NF-*κ*B signalling and proinflammatory cytokine expression [166]. There is further evidence to suggest that the anti-inflammatory properties of curcumin may have cardioprotective effects in cardiac progenitor cells [167] and augment lung maturation in fetal rats via blockade of TGF-*β* [168]. Therefore, curcumin appears to be an effective anti-inflammatory strategy in the context of obesity; despite its safety, optimal therapeutic dose and benefits in the context of obesity during pregnancy have yet to be validated* in vivo* in animals and humans.

### 6.4. Taurine

Taurine is a nonessential sulfated amino acid with a range of physiological benefits in heart function, hypertension, neuromodulation of the central nervous system, and retina function [169, 170]. Taurine is found in high amounts in mammalian plasma and cells, with a particularly high concentration in human neutrophils, where it can react with myeloperoxidase and form taurine chloramine (TauCl), which has reported anti-inflammatory effects [171]. In inflammatory conditions, taurine has been shown to decrease inflammation by downregulating NF-*κ*B [172, 173]. TauCl has been shown to oxidize I*κ*B-*α*, preventing the activation of NF-*κ*B [174].

In a human double-blind placebo controlled study, obese individuals had 41% lower plasma taurine levels compared to matched controls at baseline [175]. Eight weeks of taurine supplementation improved inflammation indices, increasing adiponectin and decreasing CRP in obese individuals. In 14 weeks of high fat feeding in mice, treatment with taurine prevented weight gain and hyperglycemia and resulted in decreased TNF*α* and IL-10 [176]. Additionally, taurine reduced macrophage infiltration and promoted shift in macrophages to an M2-like phenotype in the adipose tissue.

In models of gestational protein restriction in rodents, supplementation of taurine had protective effects on the programmed impairments to the pancreas, liver, and skeletal muscle associated with protein restriction [177–179]. Additionally, taurine was shown to normalize the changes in gene expression associated with protein restriction [177–179]. However, in the context of obesity, less is known of taurine's effect on developmental programming. A study by Li et al. showed conflicting results of taurine supplementation as a potential strategy to reverse maternal obesity-induced developmental programming effects on offspring [67]. Dams were fed an obesogenic diet (high fat: high fructose diet), which led to increased weight, hyperglycemia, insulin resistance, hepatic steatosis, and systemic inflammation. Taurine supplementation attenuated systemic inflammation, yet exacerbated impairments to lipid metabolism and inflammatory markers in the liver. In contrast, the neonates of taurine supplemented obesogenic diet dams demonstrated normalization of the detrimental hepatic proinflammatory effects of maternal obesogenic diet. In control pregnancies, taurine increased neonatal mortality and resulted in significantly lower birth weights in female pup birth weight. Although taurine supplementation did have beneficial effects in reversing programming in some conditions, further investigation is required to elucidate mechanisms of how taurine functions in the context of maternal obesity.

## 7. Other Avenues for Intervention

There is also accumulating support for the role of epigenetic regulation of gene expression as a mediator of the programming of adult-onset metabolic disease. Epigenetic regulation describes stable and heritable DNA alterations that do not involve DNA mutation including DNA methylation, posttranslational histone modifications, and chromatin remodeling [180]. Understanding how these epigenetic changes alter the postnatal phenotype could allow identification of biomarkers to enable early detection of children at risk of developing adult disease from developmental programming.

A cross-sectional study in healthy pregnant women found a positive correlation between maternal BMI and the degree of PGC-1*α* (peroxisome proliferator-activated receptor gamma coactivator 1-alpha) methylation in the umbilical cord of offspring, highlighting a potential role of DNA methylation as a mediator for the programming effects of maternal obesity [181]. Maternal obesity has been shown to induce epigenetic modifications in offspring. For example, offspring from obese mice have enhanced expression of Zfp423, accompanied by reduced methylation in the Zfp423 promoter [182]. Zfp423 is a transcription factor that plays roles in cell commitment to the adipogenic lineage; therefore these changes are likely to contribute to enhanced adipogenic differentiation during fetal development and predisposition to obesity. In a multigenerational mouse model, Ding et al. found that high fat feeding caused a “feed-forward cycle” exacerbating adipose tissue inflammatory processes via DNA hypomethylation, resulting in epigenetic changes to expression of Tlr1 and Tlr2 [183]. Maternal supplementation with methyl donors has been shown to protect offspring against the adverse effects of a maternal obesogenic diet, but whether these changes are mediated in part by alterations in inflammatory profiles is not known [184, 185]. DNA methyltransferase (DNMT3b) plays an important role in regulation of macrophage polarization through epigenetic processes. In obesity, elevations in saturated fatty acids increase DNMT3b expression, leading to DNA methylation at the PPAR-*γ*1 promoter; this may contribute to deregulated adipose tissue macrophage polarisation, inflammation, and insulin resistance [186]. Collectively, these studies demonstrate the potential of epigenetic regulation as another target for intervention to prevent or treat maternal obesity programming effects.

## 8. Discussion and Conclusion

In recent years, understanding of the developmental programming effects of maternal obesity on offspring metabolic health has expanded. However, deciphering the complex interactions and mechanistic pathways involved in the process still remains a challenge. Studies range in duration, model of obesity (cafeteria diet, high fat, and high salt/high fat), and stage of development of the intervention (i.e., periconception, gestation, lactation, and weaning). The maternal-fetal obesity paradigm is extremely complex, with factors related to overnutrition, obesity, and inflammatory processes likely impacting the development of the fetus. Additionally, a majority of studies investigating the programming effects of maternal obesity observe more pronounced impairments in male offspring, and it is not well understood why these sex-specific differences occur. Nutritional intervention remains a promising therapeutic target to minimize complications to fetal development in a poor maternal environment. However, it is unclear whether these compounds are beneficial by directly affecting offspring, or rather improving the metabolic profile in the mother. However, what is clear is that weight loss and specific dietary interventions such as decreased intake of saturated fat in women who intend to become pregnant are the most effective and safe way to improve metabolic outcomes for offspring. In conclusion, evidence from animal and clinical studies provides strong evidence for the developmental origins of obesity and metabolic disorders. Intervention strategies to ameliorate the negative outcomes of maternal obesity on offspring are greatly needed as they present an easy cost effective way of decreasing potential noncommunicable disease risk for future generations to come.
